# Socioeconomic Inequalities in Total and Site-Specific Cancer Incidence in Germany: A Population-Based Registry Study

**DOI:** 10.3389/fonc.2018.00402

**Published:** 2018-09-25

**Authors:** Jens Hoebel, Lars E. Kroll, Julia Fiebig, Thomas Lampert, Alexander Katalinic, Benjamin Barnes, Klaus Kraywinkel

**Affiliations:** ^1^Division of Social Determinants of Health, Department of Epidemiology and Health Monitoring, Robert Koch Institute, Berlin, Germany; ^2^German Centre for Cancer Registry Data, Department of Epidemiology and Health Monitoring, Robert Koch Institute, Berlin, Germany; ^3^Institute for Social Medicine and Epidemiology, University of Lübeck, Lübeck, Germany

**Keywords:** cancer registry data, socioeconomic factors, health inequalities, social class, cancer epidemiology

## Abstract

Most chronic diseases follow a socioeconomic gradient with higher rates in lower socioeconomic groups. A growing body of research, however, reveals cancer to be a disease group with very diverse socioeconomic patterning, even demonstrating reverse socioeconomic gradients for certain cancers. To investigate this matter at the German national level for the first time, this study examined socioeconomic inequalities in cancer incidence in Germany, both for all cancers combined as well as for common site-specific cancers. Population-based data on primary cancers newly diagnosed in 2010–2013 was obtained from the German Centre for Cancer Registry Data. Socioeconomic position was assessed at the district level using the German Index of Socioeconomic Deprivation, which is a composite index of area-based socioeconomic indicators. Absolute and relative socioeconomic inequalities in total and site-specific cancer incidence were analyzed using multilevel Poisson regression models with the logarithm of the number of residents as an offset. Among men, socioeconomic inequalities in cancer incidence with higher rates in more deprived districts were found for all cancers combined and various site-specific cancers, most pronounced for cancers of the lung, oral and upper respiratory tract, stomach, kidney, and bladder. Among women, higher rates in more deprived districts were evident for kidney, bladder, stomach, cervical, and liver cancer as well as for lymphoid/hematopoietic neoplasms, but no inequalities were evident for all cancers combined. Reverse gradients with higher rates in less deprived districts were found for malignant melanoma and thyroid cancer in both sexes, and in women additionally for female breast and ovarian cancer. Whereas in men the vast majority of all incident cancers occurred at cancer sites showing higher incidence rates in more deprived districts and cancers with a reverse socioeconomic gradient were in a clear minority, the situation was more balanced for women. This is the first national study from Germany examining socioeconomic inequalities in total and site-specific cancer incidence. The findings demonstrate that the socioeconomic patterning of cancer is diverse and follows different directions depending on the cancer site. The area-based cancer inequalities found suggest potentials for population-based cancer prevention and can help develop local strategies for cancer prevention and control.

## Introduction

With close to 480,000 incident cases in 2014 and causing approximately 25% of all deaths, cancer is a major health concern in Germany, as in practically all countries with high life expectancies. Although age-specific and standardized mortality rates for total cancer have been steadily declining since the mid-1990s and incidence rates, at least for men, have been showing a modest decrease in recent years, the absolute burden of cancer is increasing due to population aging.

Social epidemiological research consistently shows that socioeconomic position is an important determinant of health and disease (1–5). The term “socioeconomic position” describes the position that an individual or group holds within a vertically structured society by referring to the social and economic factors that influence this position, mainly education, employment, and income (6, 7). Previous research indicates that socioeconomic position exerts its effects on health through various pathways. For instance, people with low socioeconomic position are more likely to be exposed to health risks in the workplace and living environment than those with higher socioeconomic position (8–11). In addition, common lifestyle-related risk factors such as tobacco smoking, physical inactivity, unhealthy diet, and obesity are each more prevalent in lower socioeconomic groups (12–17). As a consequence, people with low socioeconomic position have an increased risk of severe and chronic health conditions, which is ultimately reflected in a higher risk of premature mortality and a lower life expectancy (3, 18–23). The Organization for Economic Co-operation and Development has recently estimated for 23 countries around the globe that, on average, the gap in life expectancy between high and low socioeconomic groups is 8 years for men and 5 years for women at the age of 25 (24). Similar gaps in longevity have also been reported for Germany (25–27).

Over recent decades, socioeconomic determinants have increasingly moved into the focus of cancer epidemiology. As early as 1997, the International Agency for Research on Cancer summarized in a report on the existing evidence that people with lower socioeconomic position tend to have higher cancer incidence than those with high socioeconomic position, although this pattern varies according to cancer site (28). Higher rates in lower socioeconomic groups, typically referred to as the socioeconomic gradient in health, have been found for a variety of cancers, e.g., for cancers of the respiratory tract, oral, and stomach cancer (29–33). A reverse socioeconomic gradient with higher incidence in upper socioeconomic groups has been reported especially for skin cancer and female breast cancer (34–37). In addition, evidence from some high-income countries shows that cancer contributes to a large proportion of the gap in mortality and life expectancy between low and high socioeconomic groups and that the proportion of the mortality gap attributable to cancer has increased overall in recent decades (38–40).

Evidence from Germany on socioeconomic inequalities in cancer incidence is still scarce, but the few studies available are largely consistent with those from other high-income countries in suggesting a strong socioeconomic patterning of cancer incidence for various cancer sites (41–44). However, the few findings from Germany are limited to certain regions, such as single German federal states, or to enrollees in one specific statutory health insurance fund, and therefore do not reflect the population as a whole. The only large-scale study from Germany was restricted to inequalities in cancer survival (45). Moreover, the existing studies from Germany have focused on relative inequalities in cancer between socioeconomic groups, whereas absolute inequalities have largely been neglected. The aims of the present study were therefore to use nationwide data (1) to analyze area-based socioeconomic gradients in the incidence of cancer overall and common site-specific cancers among men and women in Germany, and (2) to examine the magnitude of absolute and relative socioeconomic inequalities in cancer incidence for various cancer sites.

## Materials and methods

### Data source and study population

The analyses were based on population-based registry data from the German Centre for Cancer Registry Data at the Robert Koch Institute. All German federal states maintain population-based cancer registries that provide nationwide assessment of incident primary cancers as well as mortality follow-up. Federal and state laws regulate registry operations and practices. The registries have been operating for various lengths of time, the oldest of which is the Saarland Cancer Registry (since 1970) and the youngest of which is the cancer registry of Baden-Württemberg (since 2009). Each registry transfers an anonymized dataset annually to the Robert Koch Institute, where the data undergo quality checks and are pooled for nationwide and regional analyses. Additionally, registration completeness is estimated by federal state, year and diagnosis group. These estimates are based on comparisons of mortality-to-incidence ratios, with established reference registries providing baseline values. For the present analyses, cancer incidence data for the years 2010 through 2013 were extracted from this pooled dataset. These years were chosen so as to include reliable data from Germany's largest federal state, North Rhine-Westphalia, which established statewide registration in 2005 and achieved good completeness shortly thereafter. For cases identified only through death certificate notification (DCO cases), the date of diagnosis was set to the date of death. Data from four federal states (Baden-Württemberg, Berlin, Hesse and Saxony-Anhalt) were excluded from the present analyses due to low completeness estimates. The included registries cover nearly 59 million residents in 317 German districts (Table 1), which is approximately 73 percent of the total resident population of Germany.

**Table 1:** Description of the study population and dataset, 2010–2013.

	**Men**	**Women**
Mean population size per year	28,757,742	29,975,640
Mean number of incident cancer cases per year^a^	191,426	171,349
Number of first-level units in the data set^b^	22,824	22,824
Number of districts (second-level units)	317	317
Mean annual number of residents per district	90,718	94,560
–Deprivation quintile 1 (least deprived)	110,404	116,060
–Deprivation quintile 2	91,499	95,032
–Deprivation quintile 3	91,173	95,091
–Deprivation quintile 4	81,593	84,771
–Deprivation quintile 5 (most deprived)	78,603	81,497
Mean deprivation score of included districts (with *SD*)	0.64 ± 0.16	0.64 ± 0.16
Mean deprivation score of excluded districts (with *SD*)	0.56 ± 0.19	0.56 ± 0.19

a *For all cancer sites (C00–C97 without C44 and C77–C79)*.

b *Product of the number of age groups (n = 18), districts (n = 317), and observation years (n = 4); SD, standard deviation*.

### Cancer sites

The population-based cancer registries in Germany classify cancer diagnoses based on both the tenth edition of the International Classification of Diseases (ICD-10) and the third edition of the International Classification of Diseases for Oncology (ICD-O-3). For the present analyses, the group of all cancers combined included primary malignant cancers without non-melanoma skin cancers (ICD-10 codes C00–C43, C45–C76, and C80–C97). Individual cancer sites and cancer site groups were defined according to the given ICD-10 codes. Bladder cancers (C67) were analyzed both including and excluding *in-situ* tumors (D09.0) and tumors of uncertain or unknown behavior (D41.4).

### Socioeconomic deprivation

**Figure 1:**
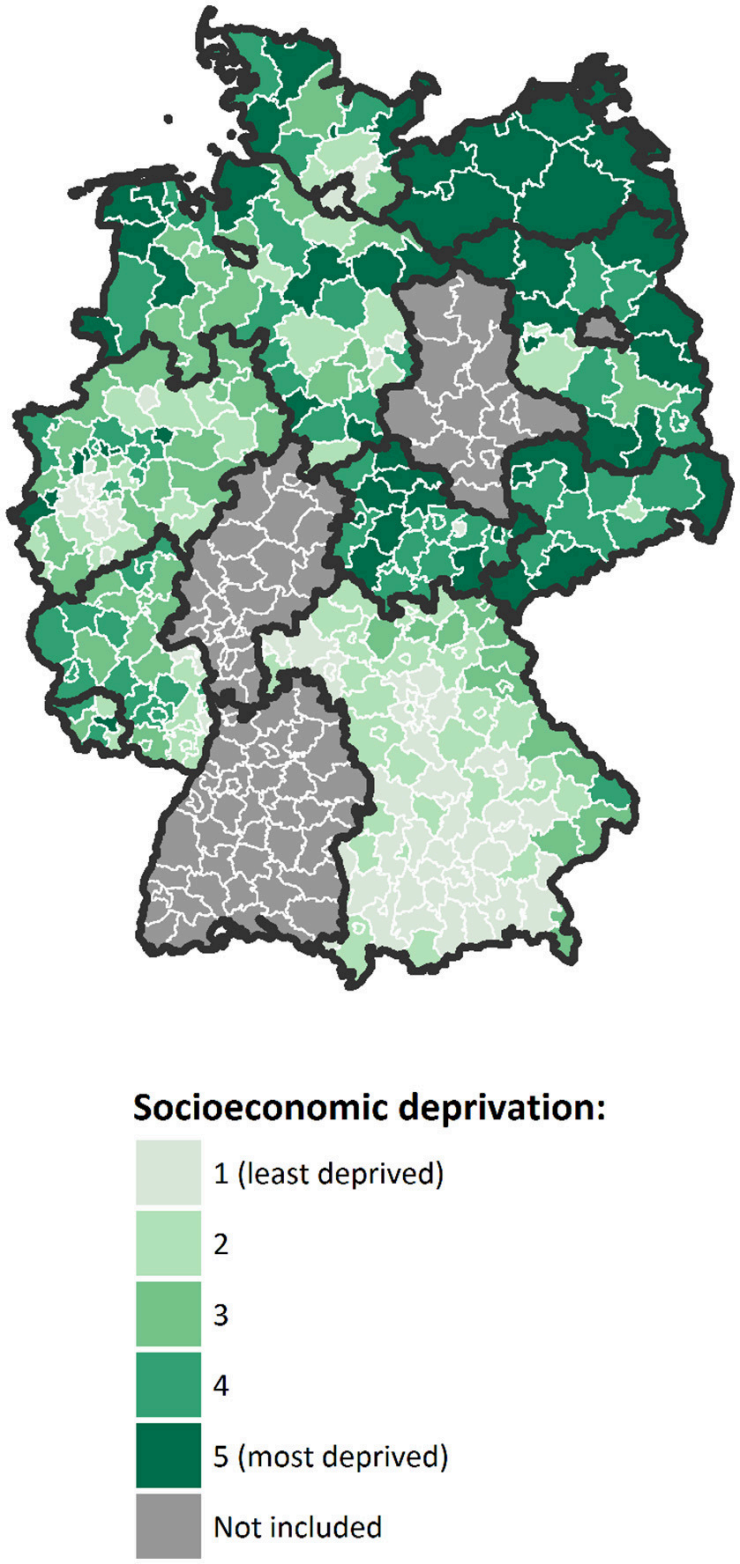
Map of Germany with districts included in the analysis, colored according to their mean level of socioeconomic deprivation over the study period, in quintiles (Geodata: © GeoBasis-DE / BKG 2018).

Area-based socioeconomic deprivation was measured at the district level using the German Index of Socioeconomic Deprivation (GISD), which has been developed by the Robert Koch Institute for epidemiological research and health reporting in Germany (46). In the present study, we used the second version of the index, which is available for research purposes free of charge at a GitHub repository (47). The index is generally available for regional units at different spatial levels. In this study, it was used at the level of German administrative districts because this was the smallest spatial level that could be analyzed in the nationally pooled cancer registry data used. GISD is a composite index with three classic socioeconomic dimensions: income, education and employment. The income dimension is assessed by area-based mean net household income, tax revenues and debtor quotas. The educational dimension is ascertained using a district's population share of employees with university degree and the share of school dropouts without certificate. In addition, during the revision of the index, the share of school graduates with Abitur (German equivalent of the International Baccalaureate) and the share of employees without any secondary-school degree have been added. Indicators of the employment dimension are the regional unemployment rate, average gross wage of employees and labor force participation rate. Factor analysis is used to weight the single indicators of each dimension. The three dimensions are then given equal weighting in the composite index, thus income, education and employment each contribute one-third to the total index score. A higher index score indicates higher socioeconomic deprivation, i.e., lower socioeconomic position of a district's population. All districts included in our analysis were classified into quintiles of socioeconomic deprivation by year according to their total index scores (Figure 1). Further information about the methods and data used in the development of the index have been published elsewhere in detail (46).

### Statistical analysis

Incidence rates with 95% confidence intervals (CI) were estimated as the number of newly diagnosed cancer cases per 100,000 residents, as predicted by Poisson regression analysis. To account for the clustered and hierarchical structure of the data (age groups nested within districts), multilevel models were used with age groups as first-level and districts as second-level units. The number of incident cancer cases registered within each age group of a district was regressed on the districts' level of socioeconomic deprivation, with age group and calendar year as covariates. Analyses were stratified by sex in order to identify sex-specific patterns of socioeconomic inequalities in cancer risk. In overall models for men and women together, sex was added to the model as an additional covariate. The logarithm of the population size in each age group was included in the models as an offset term to account for the variable number of persons under observation. To obtain age-standardized results, each district's age distribution was weighted to match the 2013 European Standard Population with a collapsed upper age band of 85+ (48). For standard error estimation, the sample size was defined as the total number of first-level units in the dataset (*n* = 22,824).

Socioeconomic inequalities in cancer incidence were analyzed by computing simple measures of pairwise group differences by quintiles of socioeconomic deprivation (standardized rate differences, standardized rate ratios) and more sophisticated summary indices of inequality (slope index of inequality [SII], relative index of inequality [RII]). While rate differences and SII quantify the magnitude of absolute inequality, rate ratios and RII represent the magnitude of relative inequality (49, 50). The advantage of the regression-based SII and RII over the simple measures is that they do not simply compare two extreme groups (e.g., the most vs. least deprived quintile), but take into account the association between a socioeconomic variable and a given health outcome across the entire socioeconomic spectrum (50). In other words, these measures make full use of all information available, whereas the simple measures ignore parts of it. To calculate the SII and RII, we used ridit scoring to convert the socioeconomic deprivation index to a fractional rank variable ranging from 0 (least deprived) to 1 (most deprived), which was then entered into the regression model as an independent variable (51, 52). The SII represents the absolute rate difference and the RII the relative rate ratio between people living in the most vs. least deprived districts. Thus, in the present study, they compare the average cancer risk between districts at the very bottom and very top of the regional socioeconomic distribution while taking into account the risk in the intermediate districts through the regression-based estimation method. Treemaps were used to visualize the proportion of all incident cancers diagnosed at each specific site in combination with the magnitude of its absolute and relative inequality.

## Results

Table 2 presents the age-standardized incidence rates for all and site-specific primary cancers, stratified by sex and quintiles of socioeconomic deprivation. Results for the total population (i.e., men and women together) and additional cancer sites can be found in the Supplementary Tables S1, S2. Among the male population, area-based socioeconomic gradients were evident for all cancers combined and a majority of the site-specific cancers considered, with higher incidence rates in the most deprived districts for cancers of the oral and upper respiratory tract, esophagus, stomach, colorectum, pancreas, lung, kidney, bladder, and lymphoid or hematopoietic neoplasms. Exceptions were malignant melanoma and thyroid cancer incidence, which followed a reverse gradient with higher rates in less deprived districts. Among the female population, socioeconomic gradients with higher rates in more deprived districts were evident for stomach, liver, cervical, kidney, bladder, and lymphoid or hematopoietic cancers. Reverse gradients with higher rates among women in less deprived districts were found for malignant melanoma, thyroid, breast and ovarian cancer. For these cancers (expect for thyroid cancer), the incidence rates among women did not show a consistent gradient, as the rates in the middle deprivation quintile tended to be higher than in the adjacent quintiles, a pattern also evident for male prostate cancer.

**Table 2:** Age-standardized incidence rates among men and women by quintiles of district-level socioeconomic deprivation.

	**Quintiles of socioeconomic deprivation**				
	**1 (least deprived)**	**2**	**3**	**4**	**5 (most deprived)**
	**SIR (95% CI)**	**SIR (95% CI)**	**SIR (95% CI)**	**SIR (95% CI)**	**SIR (95% CI)**
**ALL CANCERS (C00**–**C97)**^a^					
Men	648.7 (635.9–661.6)	664.9 (653.8–676.1)	686.8 (675.8–697.8)	691.8 (680.8–702.8)	695.9 (683.7–708.0)
Women	491.3 (480.7–501.8)	488.5 (479.6–497.3)	497.3 (488.6–505.9)	489.2 (480.7–497.8)	486.8 (477.5–496.1)
**ORAL AND UPPER RESPIRATORY TRACT (C00–C06, C09–C14, C32)**					
Men	27.3 (25.9–28.6)	27.8 (26.5–29.1)	28.6 (27.3–29.9)	32.6 (31.2–34.0)	33.9 (32.3–35.4)
Women	7.8 (7.3–8.4)	7.8 (7.3–8.4)	8.0 (7.5–8.5)	7.6 (7.1–8.2)	8.3 (7.7–8.9)
**ESOPHAGUS (C15)**					
Men	11.9 (11.3–12.6)	12.9 (12.2–13.6)	13.6 (12.9–14.3)	13.3 (12.6–13.9)	14.2 (13.5–14.9)
Women	3.1 (2.8–3.4)	3.0 (2.7–3.2)	3.3 (3.0–3.5)	3.0 (2.7–3.3)	2.8 (2.5–3.0)
**STOMACH (C16)**					
Men	23.7 (22.8–24.7)	24.5 (23.6–25.5)	25.9 (24.9–26.9)	27.4 (26.4–28.4)	29.0 (27.9–30.0)
Women	12.6 (12.0–13.1)	13.7 (13.1–14.3)	13.5 (13.0–14.1)	14.0 (13.5–14.6)	14.7 (14.1–15.3)
**COLORECTUM (C18–C20)**					
Men	88.1 (85.5–90.7)	92.2 (89.6–94.8)	93.8 (91.3–96.4)	94.3 (91.8–96.8)	94.5 (91.9–97.1)
Women	57.1 (55.3–59.0)	58.7 (56.9–60.5)	61.8 (60.0–63.6)	59.4 (57.6–61.1)	58.9 (57.1–60.7)
**LIVER (C22)**					
Men	16.6 (15.5–17.7)	15.6 (14.6–16.5)	14.9 (14.0–15.8)	16.4 (15.4–17.4)	16.9 (15.9–17.9)
Women	5.0 (4.7–5.3)	5.2 (4.9–5.6)	4.8 (4.5–5.2)	5.3 (5.0–5.7)	5.8 (5.4–6.1)
**PANCREAS (C25)**					
Men	22.5 (21.6–23.3)	21.2 (20.4–22.0)	21.6 (20.8–22.4)	22.5 (21.7–23.3)	24.0 (23.1–24.8)
Women	17.7 (17.2–18.3)	17.4 (16.8–18.0)	16.8 (16.3–17.4)	17.1 (16.5–17.6)	17.4 (16.8–18.0)
**LUNG (C33–C34)**					
Men	77.4 (74.1–80.8)	85.1 (81.9–88.2)	93.1 (89.8–96.4)	99.9 (96.4–103.3)	103.1 (99.2–106.9)
Women	35.5 (33.1–37.9)	35.5 (33.5–37.6)	38.8 (36.7–41.0)	38.6 (36.4–40.7)	37.7 (35.4–40.0)
**MALIGNANT MELANOMA OF SKIN (C43)**					
Men	31.6 (29.8–33.4)	30.0 (28.4–31.6)	28.4 (26.9–29.9)	25.0 (23.6–26.3)	22.9 (21.6–24.2)
Women	26.8 (25.0–28.7)	24.7 (23.2–26.2)	26.1 (24.6–27.6)	22.4 (21.0–23.7)	21.1 (19.6–22.5)
**BREAST (C50)**					
Women	159.1 (154.6–163.7)	156.7 (152.7–160.6)	158.5 (154.6–162.3)	152.6 (148.9–156.3)	151.7 (147.8–155.7)
**CERVIX UTERI (C53)**					
Women	10.9 (10.3–11.5)	10.7 (10.1–11.3)	11.3 (10.7–11.9)	12.5 (11.8–13.1)	12.1 (11.5–12.8)
**OVARY (C56)**					
Women	17.1 (16.4–17.8)	17.3 (16.6–18.0)	17.6 (16.9–18.3)	16.6 (15.9–17.3)	15.9 (15.2–16.5)
**PROSTATE (C61)**					
Men	168.8 (163.0–174.7)	170.8 (165.6–175.9)	178.8 (173.6–183.9)	170.6 (165.8–175.5)	173.1 (167.7–178.5)
**KIDNEY (C64)**					
Men	23.3 (22.2–24.5)	24.8 (23.6–26.0)	24.6 (23.5–25.7)	27.2 (26.0–28.4)	28.3 (27.1–29.6)
Women	11.1 (10.5–11.7)	12.1 (11.4–12.7)	12.1 (11.5–12.8)	13.2 (12.6–13.9)	14.3 (13.6–15.0)
**BLADDER (C67)**					
Men	27.7 (26.2–29.2)	29.4 (27.9–30.9)	30.9 (29.4–32.4)	31.9 (30.4–33.5)	33.2 (31.5–34.8)
Women	7.7 (7.2–8.2)	8.0 (7.5–8.6)	8.4 (7.9–8.9)	8.6 (8.1–9.1)	9.1 (8.6–9.7)
**THYROID GLAND (C73)**					
Men	6.0 (5.5–6.5)	5.0 (4.5–5.4)	5.1 (4.7–5.5)	4.0 (3.6–4.3)	3.8 (3.5–4.2)
Women	12.4 (11.4–13.4)	11.4 (10.6–12.3)	10.5 (9.7–11.3)	8.4 (7.8–9.1)	9.5 (8.7–10.2)
**LYMPHOID AND HEMATOPOIETIC NEOPLASMS (C81–C96)**					
Men	51.3 (49.3–53.3)	54.4 (52.4–56.4)	54.3 (52.4–56.2)	55.3 (53.4–57.3)	56.3 (54.3–58.4)
Women	33.7 (32.4–35.0)	35.9 (34.6–37.3)	36.5 (35.2–37.8)	37.0 (35.7–38.3)	37.6 (36.2–38.9)

a *Without C44 and C77–C79; CI, confidence interval; SIR, standardized incidence rate; All rates are predictive margins (predicted cases per 100,000 residents) from multilevel Poisson regression models, weighted according to the 2013 European Standard Population*.

Table 3 shows the measures of absolute inequalities (standardized rate differences and SII) and relative inequalities (standardized rate ratios and RII) in primary cancer incidence by sex. Results for the total population and additional cancer sites can be found in Supplementary Table S3. For the overall category of all primary cancers, the age-standardized incidence rate for men living in the most deprived quintile of districts was 47 cases per 100,000 residents higher compared to their counterparts in the least deprived quintile. This rate difference was even larger (71 cases per 100,000 residents) when districts at the very top and very bottom of the regional socioeconomic spectrum were compared and the distribution between them was taken into account, as indicated by the SII. With regard to relative inequalities, the standardized incidence rate was 7% higher among men in the most deprived quintile compared to their counterparts in the least deprived quintile. Again, the increase was larger (11%) when the entire socioeconomic distribution was taken into account, as reflected in the RII. Among men, lung cancer and malignant melanoma showed the highest magnitudes of absolute inequalities, although their socioeconomic inequalities followed different patterns, with higher rates in more deprived districts for lung cancer and a reverse gradient for malignant melanoma. The largest relative inequalities among men were found for thyroid cancer, malignant melanoma, lung cancer, and cancers of the oral and upper respiratory tract.

**Table 3:** Absolute and relative inequalities in cancer incidence among men and women by district-level socioeconomic deprivation.

	**Simple measures**			**Summary indices**		
	**SRD (95% CI)**	**SRR (95% CI)**	***p*-value**	**SII (95% CI)**	**RII (95% CI)**	***p*-value**
**ALL CANCERS (C00–C97)**^a^						
Men	47.1 (29.7–64.6)	1.07 (1.05–1.10)	<0.001	71.3 (47.8–94.9)	1.11 (1.07–1.15)	<0.001
Women	−4.5 (−18.3–9.4)	0.99 (0.96–1.02)	0.526	−7.5 (−26.4–11.5)	0.98 (0.95–1.02)	0.440
**ORAL AND UPPER RESPIRATORY TRACT (C00–C06, C09–C14, C32)**						
Men	6.6 (4.5–8.6)	1.24 (1.16–1.33)	<0.001	9.8 (7.3–12.2)	1.38 (1.28–1.50)	<0.001
Women	0.5 (−0.3–1.3)	1.06 (0.96–1.17)	0.246	0.4 (−0.5–1.3)	1.05 (0.94–1.17)	0.403
**ESOPHAGUS (C15)**						
Men	2.3 (1.3–3.2)	1.19 (1.11–1.28)	<0.001	2.5 (1.4–3.6)	1.21 (1.11–1.31)	<0.001
Women	−0.3 (−0.7–0.1)	0.90 (0.79–1.02)	0.106	−0.3 (−0.8–0.1)	0.89 (0.77–1.04)	0.141
**STOMACH (C16)**						
Men	5.2 (3.8–6.6)	1.22 (1.16–1.29)	<0.001	6.7 (5.1–8.2)	1.29 (1.22–1.37)	<0.001
Women	2.2 (1.3–3.0)	1.17 (1.10–1.24)	<0.001	2.4 (1.5–3.3)	1.19 (1.12–1.27)	<0.001
**COLORECTUM (C18–C20)**						
Men	6.4 (2.8–10.1)	1.07 (1.03–1.12)	0.001	8.0 (3.6–12.4)	1.09 (1.04–1.14)	<0.001
Women	1.7 (−0.9–4.3)	1.03 (0.99–1.08)	0.189	2.5 (−0.6–5.7)	1.04 (0.99–1.10)	0.115
**LIVER (C22)**						
Men	0.3 (−1.2–1.7)	1.02 (0.93–1.11)	0.714	0.4 (−1.3–2.1)	1.03 (0.92–1.14)	0.632
Women	0.8 (0.3–1.3)	1.16 (1.06–1.27)	0.001	0.8 (0.3–1.4)	1.17 (1.06–1.29)	0.003
**PANCREAS (C25)**						
Men	1.5 (0.3–2.7)	1.07 (1.01–1.12)	0.015	1.9 (0.6–3.3)	1.09 (1.03–1.16)	0.005
Women	−0.3 (−1.1–0.5)	0.98 (0.94–1.03)	0.477	−0.4 (−1.3–0.5)	0.98 (0.93–1.03)	0.379
**LUNG (C33–C34)**						
Men	25.6 (20.5–30.7)	1.33 (1.26–1.41)	<0.001	37.1 (30.5–43.7)	1.50 (1.40–1.61)	<0.001
Women	2.2 (−1.0–5.5)	1.06 (0.97–1.16)	0.183	2.7 (−1.7–7.0)	1.07 (0.96–1.21)	0.232
**MALIGNANT MELANOMA OF SKIN (C43)**						
Men	−8.7 (−10.9–−6.5)	0.72 (0.67–0.79)	<0.001	−12.3 (−15.0–−9.5)	0.64 (0.58–0.71)	<0.001
Women	−5.8 (−8.1–−3.5)	0.78 (0.71–0.86)	<0.001	−8.3 (−11.3–−5.4)	0.71 (0.63–0.80)	<0.001
**BREAST (C50)**						
Women	−7.4 (−13.4–−1.4)	0.95 (0.92–0.99)	0.016	−12.0 (−19.6–−4.4)	0.93 (0.88–0.97)	0.002
**CERVIX UTERI (C53)**						
Women	1.3 (0.4–2.2)	1.12 (1.03–1.21)	0.005	2.0 (1.0–3.0)	1.19 (1.09–1.30)	<0.001
**OVARY (C56)**						
Women	−1.2 (−2.2–−0.2)	0.93 (0.87–0.99)	0.016	−1.7 (−2.9–−0.6)	0.90 (0.84–0.97)	0.003
**PROSTATE (C61)**						
Men	4.3 (−3.6–12.2)	1.03 (0.98–1.07)	0.286	2.6 (−7.7–12.9)	1.02 (0.96–1.08)	0.623
**KIDNEY (C64)**						
Men	5.0 (3.3–6.6)	1.21 (1.14–1.29)	<0.001	6.3 (4.3–8.3)	1.28 (1.18–1.38)	<0.001
Women	3.2 (2.2–4.1)	1.28 (1.19–1.38)	<0.001	4.1 (3.1–5.2)	1.39 (1.28–1.51)	<0.001
**BLADDER (C67)**						
Men	5.5 (3.3–7.7)	1.20 (1.11–1.29)	<0.001	7.4 (4.7–10.0)	1.27 (1.17–1.39)	<0.001
Women	1.4 (0.6–2.2)	1.18 (1.08–1.29)	<0.001	1.6 (0.7–2.4)	1.21 (1.09–1.34)	<0.001
**THYROID GLAND (C73)**						
Men	−2.2 (−2.8–−1.6)	0.64 (0.57–0.72)	<0.001	−2.8 (−3.4–−2.1)	0.56 (0.49–0.64)	<0.001
Women	−3.0 (−4.2–−1.7)	0.76 (0.68–0.85)	<0.001	−5.1 (−6.5–−3.6)	0.62 (0.54–0.70)	<0.001
**LYMPHOID AND HEMATOPOIETIC NEOPLASMS (C81–C96)**						
Men	5.0 (2.2–7.9)	1.10 (1.04–1.16)	0.001	5.5 (2.1–9.0)	1.11 (1.04–1.18)	0.002
Women	3.8 (2.0–5.7)	1.11 (1.06–1.17)	<0.001	4.2 (2.0–6.5)	1.12 (1.06–1.20)	<0.001

a *Without C44 and C77–C79; SRD, standardized rate difference (most vs. least deprived quintile); SRR, standardized rate ratio (most vs. least deprived quintile); SII, Slope index of inequality; RII, relative index of inequality; CI, confidence interval. All measures are age–standardized according to the 2013 European Standard Population*.

Among women, neither absolute nor relative inequalities were found for the overall category of all primary cancers. When considering site-specific incidence, however, socioeconomic inequalities of varying degree and direction were evident for many cancer types. The largest absolute inequalities—regardless of the direction—were found for breast cancer and malignant melanoma, both of which showed a lower incidence in the most deprived districts. The largest relative inequalities among women were evident for thyroid cancer, malignant melanoma, and kidney cancer, with the latter showing higher rates in more deprived districts.

**Figure 2:**
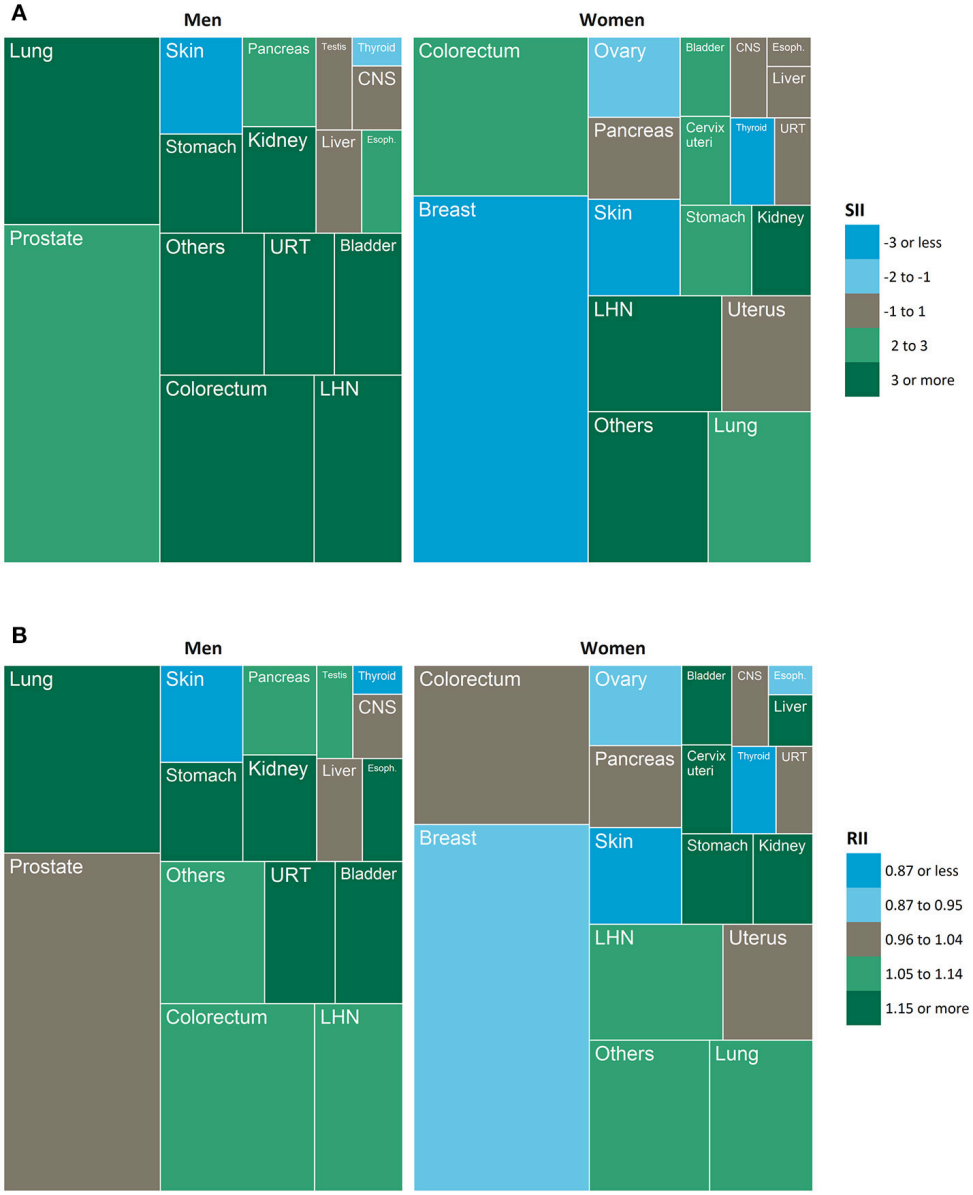
Treemaps showing the proportion of all incident cancers diagnosed at each specific site (box size) in combination with the magnitude and direction of a) absolute inequality and b) relative inequality (color). SII, slope index of inequality, RII, relative index of inequality; CNS, central nervous system; URT, upper respiratory tract; LHN, lymphoid and hematopoietic neoplasms; Esoph., esophagus.

The treemaps in Figure 2 visualize the proportion of incident primary cancers diagnosed at each specific site over the study period in combination with the pattern of its absolute and relative inequality by socioeconomic deprivation. In men, 90.1% of all incident cancers occurred at cancer sites showing absolute inequalities to the detriment of the most deprived (green); 4.5% at cancer sites with absolute inequalities to the detriment of the least deprived (blue). In women, these proportions were 46.5 and 40.3%. In respect of relative inequalities, 66.5% of all cancers in men arose at sites with relative inequalities to the detriment of the most deprived (green); 4.5% were cancer sites with relative inequalities to the detriment of the least deprived (blue). For women, these proportions were 35.8% and 40.9%, reflecting that breast and ovarian cancer with their reverse gradients made up a large proportion of cancer incidence in women.

## Discussion

### Main findings

To our knowledge, this is the first study using representative data for the vast majority of Germany's population to examine area-based socioeconomic inequalities in the incidence of cancer overall and for a variety of site-specific cancers. For all cancers combined, we found a socioeconomic gradient with higher incidence rates in more deprived districts for men but not for women. A closer look at site-specific cancers, though, showed that in both men and women, socioeconomic inequalities in cancer incidence do exist for several cancer sites, although their patterns differ between cancer sites, especially in women. The majority of cancers among men followed a socioeconomic gradient on both the absolute and relative scale with higher rates in more deprived districts, most pronounced for cancers of the lung, oral and upper respiratory tract, stomach, kidney, and bladder. Among women, this pattern was found for cancers of the kidney, bladder, stomach, and cervix uteri. Malignant melanoma of the skin and thyroid cancer were exceptions in both sexes as they showed a reverse socioeconomic gradient with the highest incidence in the least deprived districts. This pattern was also observed for female breast cancer and less clearly for ovarian cancer. Whereas in men, most incident cancers were diagnosed at sites showing a gradient to the detriment of the most deprived, in women the shares of incident cancers showing this gradient and of those with a reverse gradient were more balanced.

### Comparison with previous research and possible explanations

Our findings support a large and growing body of evidence indicating for various countries that the incidence of cancer is unequally distributed across socioeconomic groups (28, 37, 53, 54). To a large extent, our results are consistent with previous findings from various countries, which have found higher incidence rates in lower socioeconomic groups for a variety of site-specific cancers, e.g., for respiratory tract, oral and stomach cancers (29–33). The reverse socioeconomic gradients in melanoma, female breast cancer and thyroid cancer have been found in other countries before as well (34–37, 55, 56). In addition, our findings largely support those of the few previous studies from Germany. Eberle et al. analyzed socioeconomic inequalities in cancer incidence in Bremen, a major city in northern Germany (42). For all cancers combined, they reported a socioeconomic gradient for men, with higher incidence rates in more deprived town districts, but not for women. Furthermore, they found higher incidence rates for tumors of the oral cavity and pharynx as well as for lung, cervical, and bladder cancers in more deprived town districts and a reverse gradient for female breast cancer, skin and prostate cancer, which is, except for prostate cancer, very much in line with our findings. Kuznetsov et al. examined area-based socioeconomic inequalities in lung and colorectal cancer incidence in Bavaria, a southern German federal state (43, 57). Their results indicate an excess risk in more deprived areas for lung cancer in men and for colorectal cancer in both men and women. Geyer used individual data from one of the German statutory health insurance funds. He found that individuals from the lowest socioeconomic group had increased risks of lung, stomach and intestinal cancer (41). For female breast cancer incidence, however, no socioeconomic gradient was evident in the statutory health insurance data. This may have been related to the fact that high socioeconomic groups are underrepresented in the German statutory health insurance, which is especially the case with the particular insurance fund considered in the study (41, 58).

The literature suggests several explanations for socioeconomic inequalities in cancer risk, including unequal distribution of lifestyle-related risk factors, occupational, and environmental exposures, reproductive and healthcare factors. In many countries around the globe, common lifestyle-related cancer risk factors such as tobacco smoking, unhealthy diets, physical inactivity, and obesity are more prevalent in lower socioeconomic groups (12–17). Accordingly, these factors are often adduced to explain the socioeconomic patterning of cancer. Tobacco smoking, for instance, has been found to explain a major part of socioeconomic inequalities in the incidence of lung cancer (59), but also has explanatory value for socioeconomic inequalities in cancers at other sites (60–62). Regarding overall cancer mortality instead of site-specific cancer incidence, smoking has been found to explain the greatest proportion of the association with area-level socioeconomic deprivation, followed by diet, physical activity, cancer screening behaviors and body-mass-index (63). The fact that we found a socioeconomic gradient in lung cancer incidence for men but not for women is probably due to differences in the evolution of the socioeconomic gradient in smoking habits by sex. Research from Germany suggests that the socioeconomic gradient in smoking among men had already developed early in the 20th century, whereas among women it emerged much later toward the end of the century (64, 65). This difference might lead to a delay of several decades before the socioeconomic gradient in lung cancer also becomes apparent in women. Alcohol consumption has also been found to partially explain socioeconomic inequalities in different cancers (60, 62, 66, 67), but its contribution may generally be smaller than that of smoking. This may partly be due to the fact that fewer cancer cases are attributable to alcohol compared to tobacco (68, 69), but also because alcohol consumption shows only minor variation across socioeconomic groups (70), which has also been found in the German population (71). Conway et al. have found that smoking, alcohol consumption and diets low in fresh fruits and vegetables together explain around two-thirds of the excess risk for upper aerodigestive tract cancer in the lowest socioeconomic group (62). From the unexplained excess risk they conclude that low socioeconomic position seems to be associated with cancer risk for reasons other than only through behavioral risk factors.

In addition to lifestyle factors, the contributions of carcinogen exposure at work and in the living environment to socioeconomic inequalities in cancer risk have also been examined (60). The findings of Menvielle et al. suggest that a substantial proportion of the socioeconomic gap in hypopharyngeal and laryngeal cancer is attributable to occupational exposures to asbestos, coal dust and formaldehyde (60). This finding was supported in a study by Santi et al. who found that exposure to potentially carcinogenic agents at work can explain approximately a quarter of the socioeconomic inequalities in laryngeal cancer (67). Another study showed that occupational exposures to asbestos, heavy metals and polycyclic aromatic hydrocarbons can explain parts of the association between socioeconomic position and lung cancer incidence among men, with asbestos making the largest contribution (72). However, it is not only the workplace where members of lower socioeconomic groups are exposed to carcinogenic agents. Exposures to air pollution in the living environment or tobacco smoke at home can also contribute to socioeconomic inequalities in the risk of respiratory tract cancers (73).

According to a systematic review (36), the reverse gradient in female breast cancer incidence may be primarily explained by reproductive factors. Women with higher socioeconomic position are more likely to be older at first birth and have lower parity, each of which is associated with increased breast cancer risk. Socioeconomic differentials in the use of hormone replacement therapy may also play a role in this context (74), and could also help explain the reverse gradient in ovarian cancer. Concerning the reverse gradient in melanoma of the skin, a systematic review suggests that lifestyle-related risk factors, including recreational sun exposure and tanning, may explain why higher socioeconomic groups show higher melanoma incidence (75).

Mechanisms related to healthcare should not be neglected when it comes to explaining socioeconomic inequalities in cancer incidence, especially when the findings are based on population-based registry data. The reverse gradient in thyroid cancer, for instance, is hypothesized to be attributable to new diagnostic capabilities, which may have led to overdiagnosis and increases in thyroid cancer incidence that have been observed in recent decades (56, 76). As with previous innovations in disease prevention and early disease detection such as the polio vaccine or the pap test (77), innovative diagnostic tools for thyroid cancer detection are likely to be used more often by people from higher socioeconomic groups—at least in the first years after launch—, consequently resulting in higher incidence rates among the better-off. Moreover, the uptake of general health checks and participation in cancer screening has often been found to vary between socioeconomic groups, usually with highest participation rates in middle or upper socioeconomic groups (78>–80). This has also been the case for Germany's cancer screening programs during our study period (81). Accordingly, socioeconomic differentials in screening participation may have contributed, at least in part, to the reverse socioeconomic gradients or peak incidence in middle socioeconomic groups we observed for certain cancers. For example, Germany introduced a nationwide skin cancer screening program in 2008, and is thus, to our knowledge, the only country worldwide with such a program on a nationwide scale (82). Melanoma incidence increased in Germany after screening was introduced (83), and screening participation has been higher in the upper socioeconomic groups (84). Therefore, screening may have contributed to our finding of a higher melanoma incidence in less deprived districts. Similarly, Germany has a nationwide mammography screening program for the early detection of female breast cancer, and regular screening participation has been found to be highest in the middle and upper socioeconomic groups (81). Mammography screening may thus also have contributed to our finding that the incidence of breast cancer was highest in the middle and at the top of the socioeconomic spectrum.

### Strengths and limitations

Our study contributes to the growing interest in analyzing the association between socioeconomic deprivation and cancer. The findings extend those from previous German studies by providing results that are nearly nationally representative, covering 73% of Germany's total population, 12 of the 16 German federal states and 317 of the 402 administrative districts. Previous studies have either been restricted to one federal state or the population of one specific statutory health insurance fund. Including the vast majority of the German population regardless of health insurance status [people with private health insurance have on average a higher socioeconomic position (58)] resulted in greater socioeconomic heterogeneity of the study population.

A strength of the socioeconomic deprivation index used in the present study is that it is based exclusively on the three core dimensions of socioeconomic inequality (education, income, and employment). This facilitates the interpretation of results (46), especially when compared to indices of multiple deprivation that include domains going beyond purely socioeconomic ones, such as social capital, the share of lone-parent households, crime rates, the physical environment or morbidity (85–87). Another advantage of the index used is its public availability, which makes the analysis more easily reproducible. Nevertheless, the composite nature of the index also has some limitations. Analogous to socioeconomic indices at the individual level, composite measures generally have the disadvantage that they can conceal variation in the associations of the single dimensions with the health outcome under study (88). For example, if education were to predict a health outcome such as cancer, income or employment might not. This possibility should be considered when interpreting our results.

It should further be considered that the area-based measure of socioeconomic deprivation is prone to misclassification of subjects when interpreting it at the individual level. The socioeconomic groups in our study were classified by an area-based index, because individual-level information on socioeconomic position was not available in the cancer registry and population data. For example, the area-based approach classifies individuals with high socioeconomic position into the socioeconomically most deprived group when they live in a district with a high share of inhabitants of low socioeconomic position. Therefore, it cannot be inferred directly from our results that individuals of low or high socioeconomic position have higher or lower cancer incidence. Depending on the degree of this misclassification, which cannot be quantified with the data used, the area-based approach may have led to an underestimation of cancer inequalities with regard to individual socioeconomic position in our study. However, the area-based approach helps to identify regions whose populations, from a public health perspective, may have an increased need for cancer prevention measures with respect to specific cancers, although the large size and heterogeneity of some districts may be challenging in this respect.

We compared current cancer incidence with current district-level socioeconomic deprivation. This does not account for changes in a district's deprivation over time, nor does it account for population migration between districts with different deprivation levels. Therefore, although the GISD is fairly stable over time [intra-class correlation (ICC_2010−2013_) = 0.989] and current socioeconomic deprivation may be associated with utilization of certain diagnostic and early detection services, our analyses do not provide a complete picture of the etiologic relevance of socioeconomic deprivation for cancer incidence. Moreover, considering that a large proportion of highly deprived districts are located in Eastern Germany, it would have been desirable to discriminate the effects of living in a currently deprived district from the effect of being born and raised in the former German Democratic Republic. Since we lacked the necessary background variables in the registry data, we were not able to gain any insights into this matter.

It should be considered that excluding data from four German federal states may have introduced bias into our results. We decided to exclude data from Hesse, Saxony-Anhalt, Baden-Württemberg and Berlin because of insufficient registration completeness (estimated completeness <90%). Therefore, the extent of any associations between socioeconomic deprivation and cancer incidence in these regions remains unknown. The districts excluded from the analysis differed from the included districts in having on average lower deprivation scores and a larger heterogeneity (see Table 1), which may have introduced potential selection bias.

Another limitation is related to the heterogeneity of the included districts. In Germany, administrative units at the district level vary considerably in their population size, population density and socioeconomic diversity. Therefore the Modifiable Area Unit Problem (MAUP) (89) has to be taken into account: the MAUP postulates that different regional aggregations of the units of observation may lead to different results and conclusions. However, it has been shown that district-level estimates of the socioeconomic gradient in health for Germany, such as those presented here, tend to find less pronounced associations than estimates at smaller levels of spatial aggregation because differences in deprivation between districts are less pronounced than, for example, differences between individual towns or neighborhoods (46). Therefore, it seems likely that our results, which are in some instances based on districts with more than a million inhabitants (large metropolitan cities forming one independent administrative district), tend to underestimate the association between socioeconomic deprivation and cancer incidence.

### Conclusions

Socioeconomic inequalities in the incidence of common cancers demonstrate potentials for population-based cancer prevention. In view of the major risk factors of common cancers and the explanatory approaches discussed, both behavioral and structural prevention strategies should be identified to reduce socioeconomic differences in morbidity and mortality. In accordance with the health-in-all-policies approach, these should be implemented not only in the health sector, but in all policy areas. The area-based cancer inequalities found in our study can help to identify districts with high rates of certain cancers and to develop local and community-based strategies for cancer prevention and control. In future studies, more in-depth analyses including additional data on tumor stage and cancer mortality could provide additional insights into the social epidemiology of cancer and potential entry points for reducing the health gap between the better- and worse-off.

## Data availability statement

The dataset supporting the conclusions of this article can be obtained upon application from the German Centre for Cancer Registry Data (ZfKD) at the Robert Koch Institute. Researchers may submit an application form and a project sketch to access the anonymized dataset. Details for acquiring the data are available at: https://www.krebsdaten.de/Krebs/EN/Content/ScientificUseFile/scientificusefile_node.html

The German Index of Socioeconomic Deprivation (GISD) used in our analysis is available for research purposes free of charge at a GitHub repository: https://lekroll.github.io/GISD.

## Ethics statement

Neither approval by the Ethics Committee nor consensus procedures were required as the analyses were based on population-based cancer registry data collected in the German federal states and transferred to the German Centre for Cancer Registry Data in accordance with state and federal laws. Additional interviews, physical examinations, biological sampling or laboratory tests were not performed.

## Author contributions

JH, LK, BB, and KK designed the study. JH performed the statistical analysis. LK and BB contributed to the statistical analysis and preparation of the data for analysis. JF and JH reviewed the literature. JH drafted the manuscript with contributions of all co-authors. TL and KK supervised the study. TL, AK, and KK reviewed the manuscript critically. All authors contributed to interpretation of findings, reviewed, edited, and approved the final manuscript.

### Conflict of interest statement

The authors declare that the research was conducted in the absence of any commercial or financial relationships that could be construed as a potential conflict of interest.
