# Supplementary material for: Socioeconomic Inequalities in Total and Site-Specific Cancer Incidence in Germany: A Population-Based Registry Study
Source: Front Oncol. 2018 Sep 25;8:402. doi: 10.3389/fonc.2018.00402 (PMC6167637; doi:10.3389/fonc.2018.00402)
Supplement: Supplementary file 1 [file Data_Sheet_1.pdf]

Supplementary Material

Socioeconomic inequalities in total and site-specific cancer incidence in Germany: a population-based registry study

Jens Hoebel*, Lars E. Kroll, Julia Fiebig, Thomas Lampert, Alexander Katalinic, Benjamin Barnes, Klaus Kraywinkel

*** Correspondence:** Jens Hoebel (j.hoebel@rki.de)

Content:

SUPPLEMENTARY TABLE S1 – Age-standardized incidence rates among men and women by quintiles of district-level socioeconomic deprivation

SUPPLEMENTARY TABLE S2 – Age-standardized incidence rates among men and women by quintiles of district-level socioeconomic deprivation

SUPPLEMENTARY TABLE S3 – Absolute and relative inequalities in cancer incidence among men and women by district-level socioeconomic deprivation

SUPPLEMENTARY TABLE S1 – Age-standardized incidence rates for cancer by sex

	Men	Women	Total
**All cancers (C00–C97)[a]**			
SIR (95% CI)	677.6 (670.4–684.7)	490.6 (484.9–496.3)	575.4 (569.5–581.2)
**Upper respiratory tract (C00–C06, C09–C14, C32)**			
SIR (95% CI)	30.0 (29.3–30.7)	7.9 (7.7–8.2)	19.0 (18.6–19.4)
**Esophagus (C15)**			
SIR (95% CI)	13.2 (12.8–13.5)	3.0 (2.9–3.1)	8.1 (7.9–8.3)
**Stomach (C16)**			
SIR (95% CI)	26.1 (25.6–26.6)	13.7 (13.4–14.0)	19.9 (19.6–20.3)
**Colorectum (C18–C20)**			
SIR (95% CI)	92.6 (91.3–93.9)	59.2 (58.3–60.1)	75.9 (74.9–76.9)
**Liver (C22)**			
SIR (95% CI)	16.1 (15.6–16.6)	5.2 (5.1–5.4)	10.7 (10.4–11.0)
**Pancreas (C25)**			
SIR (95% CI)	22.3 (21.9–22.7)	17.3 (17.0–17.5)	19.9 (19.7–20.2)
**Lung (C33–C34)**			
SIR (95% CI)	91.7 (89.7–93.6)	37.2 (35.9–38.5)	63.2 (61.7–64.8)
**Malignant melanoma of skin (C43)**			
SIR (95% CI)	27.6 (26.8–28.4)	24.2 (23.4–25.1)	25.3 (24.5–26.1)
**Breast (C50)**			
SIR (95% CI)	-	155.7 (153.5–158.0)	-
**Cervix uteri (C53)**			
SIR (95% CI)	-	11.5 (11.2–11.8)	-
**Uterus (C54-C55)**			
SIR (95% CI)	-	24.2 (23.7–24.7)	-
**Ovary (C56)**			
SIR (95% CI)	-	16.9 (16.6–17.2)	-
**Prostate (C61)**			
SIR (95% CI)	172.4 (169.3–175.5)	-	-
**Testis (C62)**			
SIR (95% CI)	10.7 (10.4–11.0)	-	-
**Kidney (C64)**			
SIR (95% CI)	25.7 (25.1–26.2)	12.6 (12.3–12.9)	19.2 (18.8–19.6)
**Bladder and other urinary sites (C65–C68)**			
SIR (95% CI)	34.9 (34.1–35.8)	10.4 (10.1–10.7)	22.5 (22.0–23.1)
**Bladder (C67)**			
SIR (95% CI)	30.6 (29.8–31.4)	8.4 (8.1–8.6)	19.4 (18.9–19.8)
**Other urinary sites (C65, C66, C68)**			
SIR (95% CI)	4.4 (4.2–4.6)	2.1 (2.0–2.2)	3.3 (3.1–3.4)
**Bladder (C67, D09.0, D41.4)**			
SIR (95% CI)	59.1 (57.7–60.4)	14.8 (14.5–15.2)	36.7 (35.8–37.5)

SUPPLEMENTARY TABLE S1 (continued)

	**Men**	**Women**	**Total**
**Bladder and other urinary sites (C65–C68, D09.0, D09.1, D41.4, D41.7)**			
SIR (95% CI)	64.5 (63.1–66.0)	17.4 (16.9–17.8)	40.6 (39.7–41.5)
**Central Nervous System (C70–C72)**			
SIR (95% CI)	10.3 (10.1–10.6)	7.2 (7.1–7.4)	8.7 (8.6–8.9)
**Thyroid gland (C73)**			
SIR (95% CI)	4.8 (4.6–5.0)	10.5 (10.0–10.9)	7.5 (7.3–7.8)
**Lymphoid/hematopoietic neoplasms (C81–C96)**			
SIR (95% CI)	54.3 (53.3–55.3)	36.2 (35.5–36.8)	45.0 (44.3–45.8)
**Others**			
SIR (95% CI)	48.4 (47.4–49.3)	40.6 (39.8–41.4)	44.2 (43.5–45.0)

[a] without C44 and C77–C79; CI, confidence interval; All rates are predictive margins (predicted cases per 100,000 residents) from multilevel Poisson regression models, weighted according to the 2013 European Standard Population.

SUPPLEMENTARY TABLE S2 – Age-standardized incidence rates among men and women by quintiles of district-level socioeconomic deprivation

	Quintiles of socioeconomic deprivation				
	**1 (least deprived)**	**2**	**3**	**4**	**5 (most deprived)**
	**SIR (95% CI)**	**SIR (95% CI)**	**SIR (95% CI)**	**SIR (95% CI)**	**SIR (95% CI)**
**All cancers (C00–C97)[a]**					
Total	565.9 (555.9–575.9)	569.7 (561.4–578.0)	582.6 (574.5–590.7)	579.8 (571.6–588.0)	578.9 (569.9–587.8)
Men	648.7 (635.9–661.6)	664.9 (653.8–676.1)	686.8 (675.8–697.8)	691.8 (680.8–702.8)	695.9 (683.7–708.0)
Women	491.3 (480.7–501.8)	488.5 (479.6–497.3)	497.3 (488.6–505.9)	489.2 (480.7–497.8)	486.8 (477.5–496.1)
**Upper respiratory tract (C00–C06, C09–C14, C32)**					
Total	17.6 (16.8–18.4)	17.9 (17.1–18.6)	18.4 (17.7–19.2)	20.1 (19.3–20.9)	20.9 (20.0–21.8)
Men	27.3 (25.9–28.6)	27.8 (26.5–29.1)	28.6 (27.3–29.9)	32.6 (31.2–34.0)	33.9 (32.3–35.4)
Women	7.8 (7.3–8.4)	7.8 (7.3–8.4)	8.0 (7.5–8.5)	7.6 (7.1–8.2)	8.3 (7.7–8.9)
**Esophagus (C15)**					
Total	7.5 (7.1–7.9)	8.0 (7.6–8.4)	8.4 (8.0–8.9)	8.2 (7.8–8.6)	8.5 (8.1–8.9)
Men	11.9 (11.3–12.6)	12.9 (12.2–13.6)	13.6 (12.9–14.3)	13.3 (12.6–13.9)	14.2 (13.5–14.9)
Women	3.1 (2.8–3.4)	3.0 (2.7–3.2)	3.3 (3.0–3.5)	3.0 (2.7–3.3)	2.8 (2.5–3.0)
**Stomach (C16)**					
Total	18.2 (17.6–18.9)	19.2 (18.6–19.9)	19.8 (19.2–20.5)	20.7 (20.0–21.3)	21.8 (21.1–22.5)
Men	23.7 (22.8–24.7)	24.5 (23.6–25.5)	25.9 (24.9–26.9)	27.4 (26.4–28.4)	29.0 (27.9–30.0)
Women	12.6 (12.0–13.1)	13.7 (13.1–14.3)	13.5 (13.0–14.1)	14.0 (13.5–14.6)	14.7 (14.1–15.3)
**Colorectum (C18–C20)**					
Total	72.9 (71.0–74.9)	75.6 (73.7–77.4)	77.7 (75.9–79.5)	76.6 (74.8–78.4)	76.8 (74.9–78.6)
Men	88.1 (85.5–90.7)	92.2 (89.6–94.8)	93.8 (91.3–96.4)	94.3 (91.8–96.8)	94.5 (91.9–97.1)
Women	57.1 (55.3–59.0)	58.7 (56.9–60.5)	61.8 (60.0–63.6)	59.4 (57.6–61.1)	58.9 (57.1–60.7)
**Liver (C22)**					
Total	10.8 (10.2–11.4)	10.5 (9.9–11.0)	9.9 (9.4–10.4)	10.9 (10.4–11.5)	11.3 (10.7–11.9)
Men	16.6 (15.5–17.7)	15.6 (14.6–16.5)	14.9 (14.0–15.8)	16.4 (15.4–17.4)	16.9 (15.9–17.9)
Women	5.0 (4.7–5.3)	5.2 (4.9–5.6)	4.8 (4.5–5.2)	5.3 (5.0–5.7)	5.8 (5.4–6.1)
**Pancreas (C25)**					
Total	20.1 (19.6–20.7)	19.4 (18.9–20.0)	19.4 (18.8–19.9)	19.9 (19.4–20.5)	20.8 (20.2–21.3)
Men	22.5 (21.6–23.3)	21.2 (20.4–22.0)	21.6 (20.8–22.4)	22.5 (21.7–23.3)	24.0 (23.1–24.8)
Women	17.7 (17.2–18.3)	17.4 (16.8–18.0)	16.8 (16.3–17.4)	17.1 (16.5–17.6)	17.4 (16.8–18.0)
**Lung (C33–C34)**					
Total	56.4 (53.9–59.0)	59.6 (57.4–61.9)	64.5 (62.2–66.8)	67.7 (65.3–70.1)	68.0 (65.4–70.7)
Men	77.4 (74.1–80.8)	85.1 (81.9–88.2)	93.1 (89.8–96.4)	99.9 (96.4–103.3)	103.1 (99.2–106.9)
Women	35.5 (33.1–37.9)	35.5 (33.5–37.6)	38.8 (36.7–41.0)	38.6 (36.4–40.7)	37.7 (35.4–40.0)
**Malignant melanoma of skin (C43)**					
Total	28.1 (26.5–29.7)	26.7 (25.3–28.0)	26.6 (25.3–27.9)	23.2 (22.1–24.4)	21.8 (20.6–23.0)
Men	31.6 (29.8–33.4)	30.0 (28.4–31.6)	28.4 (26.9–29.9)	25.0 (23.6–26.3)	22.9 (21.6–24.2)
Women	26.8 (25.0–28.7)	24.7 (23.2–26.2)	26.1 (24.6–27.6)	22.4 (21.0–23.7)	21.1 (19.6–22.5)
**Breast (C50)**					
Total	-	-	-	-	-
Men	-	-	-	-	-
Women	159.1 (154.6–163.7)	156.7 (152.7–160.6)	158.5 (154.6–162.3)	152.6 (148.9–156.3)	151.7 (147.8–155.7)

SUPPLEMENTARY TABLE S2 (continued)

	Quintiles of socioeconomic deprivation				
	**1 (least deprived)**	**2**	**3**	**4**	**5 (most deprived)**
	**SIR (95% CI)**	**SIR (95% CI)**	**SIR (95% CI)**	**SIR (95% CI)**	**SIR (95% CI)**
**Cervix uteri (C53)**					
Total	-	-	-	-	-
Men	-	-	-	-	-
Women	10.9 (10.3–11.5)	10.7 (10.1–11.3)	11.3 (10.7–11.9)	12.5 (11.8–13.1)	12.1 (11.5–12.8)
**Uterus (C54–C55)**					
Total	-	-	-	-	-
Men	-	-	-	-	-
Women	23.9 (23.0–24.9)	24.4 (23.4–25.4)	24.2 (23.3–25.2)	24.4 (23.5–25.4)	24.0 (23.1–25.0)
**Ovary (C56)**					
Total	-	-	-	-	-
Men	-	-	-	-	-
Women	17.1 (16.4–17.8)	17.3 (16.6–18.0)	17.6 (16.9–18.3)	16.6 (15.9–17.3)	15.9 (15.2–16.5)
**Prostate (C61)**					
Total	-	-	-	-	-
Men	168.8 (163.0–174.7)	170.8 (165.6–175.9)	178.8 (173.6–183.9)	170.6 (165.8–175.5)	173.1 (167.7–178.5)
Women	-	-	-	-	-
**Testis (C62)**					
Total	-	-	-	-	-
Men	10.1 (9.5–10.7)	10.6 (10.0–11.3)	11.0 (10.4–11.6)	11.0 (10.4–11.6)	10.6 (9.9–11.2)
Women	-	-	-	-	-
**Kidney (C64)**					
Total	17.4 (16.7–18.2)	18.6 (17.8–19.3)	18.6 (17.9–19.4)	20.4 (19.6–21.1)	21.1 (20.3–22.0)
Men	23.3 (22.2–24.5)	24.8 (23.6–26.0)	24.6 (23.5–25.7)	27.2 (26.0–28.4)	28.3 (27.1–29.6)
Women	11.1 (10.5–11.7)	12.1 (11.4–12.7)	12.1 (11.5–12.8)	13.2 (12.6–13.9)	14.3 (13.6–15.0)
**Bladder and other urinary sites (C65–C68)**					
Total	20.6 (19.6–21.6)	21.8 (20.8–22.8)	22.7 (21.7–23.7)	23.5 (22.5–24.5)	24.2 (23.1–25.3)
Men	31.8 (30.1–33.4)	33.6 (31.9–35.2)	35.2 (33.6–36.9)	36.5 (34.8–38.2)	37.7 (35.9–39.4)
Women	9.6 (9.0–10.1)	10.2 (9.5–10.8)	10.6 (10.0–11.2)	10.6 (10.0–11.2)	11.2 (10.6–11.9)
**Bladder (C67)**					
Total	17.6 (16.7–18.5)	18.6 (17.7–19.5)	19.4 (18.6–20.3)	20.2 (19.3–21.1)	20.9 (20.0–21.9)
Men	27.7 (26.2–29.2)	29.4 (27.9–30.9)	30.9 (29.4–32.4)	31.9 (30.4–33.5)	33.2 (31.5–34.8)
Women	7.7 (7.2–8.2)	8.0 (7.5–8.6)	8.4 (7.9–8.9)	8.6 (8.1–9.1)	9.1 (8.6–9.7)
**Other urinary sites (C65, C66, C68)**					
Total	3.0 (2.8–3.3)	3.2 (3.0–3.5)	3.4 (3.1–3.6)	3.3 (3.0–3.5)	3.3 (3.1–3.6)
Men	4.3 (3.8–4.7)	4.4 (4.0–4.8)	4.5 (4.1–4.9)	4.6 (4.2–5.0)	4.5 (4.1–4.9)
Women	1.9 (1.7–2.1)	2.2 (1.9–2.4)	2.3 (2.1–2.5)	2.0 (1.8–2.2)	2.2 (1.9–2.4)
**Bladder (C67, D09.0, D41.4)**					
Total	33.4 (31.9–34.9)	35.9 (34.5–37.4)	37.9 (36.4–39.4)	38.0 (36.6–39.5)	38.1 (36.6–39.7)
Men	53.4 (50.9–55.9)	57.5 (55.1–60.0)	61.3 (58.8–63.8)	61.8 (59.3–64.2)	61.5 (58.9–64.1)
Women	13.7 (12.9–14.5)	14.5 (13.7–15.3)	15.4 (14.6–16.2)	15.0 (14.2–15.8)	15.6 (14.8–16.4)

SUPPLEMENTARY TABLE S2 (continued)

	Quintiles of socioeconomic deprivation				
	**1 (least deprived)**	**2**	**3**	**4**	**5 (most deprived)**
	**SIR (95% CI)**	**SIR (95% CI)**	**SIR (95% CI)**	**SIR (95% CI)**	**SIR (95% CI)**
**Bladder and other urinary sites (C65–C68, D09.0, D09.1, D41.4, D41.7)**					
Total	37.2 (35.5–38.8)	39.9 (38.3–41.5)	42.0 (40.4–43.6)	41.9 (40.4–43.5)	42.2 (40.5–43.9)
Men	58.5 (55.8–61.3)	62.9 (60.3–65.5)	66.7 (64.1–69.4)	67.2 (64.6–69.8)	67.3 (64.5–70.1)
Women	16.0 (15.1–16.9)	17.0 (16.1–17.9)	18.2 (17.2–19.1)	17.5 (16.6–18.3)	18.1 (17.2–19.1)
**Central Nervous System (C70–C72)**					
Total	8.7 (8.4–9.1)	8.6 (8.2–9.0)	8.7 (8.4–9.1)	8.8 (8.5–9.2)	8.8 (8.5–9.2)
Men	10.4 (9.9–10.8)	10.1 (9.6–10.6)	10.4 (9.9–10.9)	10.5 (10.0–11.0)	10.3 (9.8–10.8)
Women	7.2 (6.8–7.6)	7.3 (6.9–7.7)	7.2 (6.8–7.6)	7.2 (6.8–7.6)	7.4 (7.0–7.8)
**Thyroid gland (C73)**					
Total	9.0 (8.4–9.6)	8.1 (7.5–8.6)	7.8 (7.3–8.3)	6.1 (5.7–6.6)	6.6 (6.1–7.1)
Men	6.0 (5.5–6.5)	5.0 (4.5–5.4)	5.1 (4.7–5.5)	4.0 (3.6–4.3)	3.8 (3.5–4.2)
Women	12.4 (11.4–13.4)	11.4 (10.6–12.3)	10.5 (9.7–11.3)	8.4 (7.8–9.1)	9.5 (8.7–10.2)
**Lymphoid/hematopoietic neoplasms (C81–C96)**					
Total	42.5 (41.0–44.0)	44.9 (43.5–46.3)	45.4 (44.0–46.8)	45.8 (44.5–47.2)	46.6 (45.1–48.1)
Men	51.3 (49.3–53.3)	54.4 (52.4–56.4)	54.3 (52.4–56.2)	55.3 (53.4–57.3)	56.3 (54.3–58.4)
Women	33.7 (32.4–35.0)	35.9 (34.6–37.3)	36.5 (35.2–37.8)	37.0 (35.7–38.3)	37.6 (36.2–38.9)
**Other cancers**					
Total	42.2 (40.7–43.7)	43.4 (42.0–44.8)	44.6 (43.2–46.0)	45.4 (44.0–46.8)	45.6 (44.1–47.1)
Men	46.1 (44.3–48.0)	47.4 (45.6–49.2)	48.8 (47.0–50.6)	49.5 (47.7–51.3)	50.1 (48.2–51.9)
Women	38.6 (37.0–40.2)	39.8 (38.3–41.4)	41.1 (39.5–42.7)	41.4 (39.8–42.9)	42.1 (40.5–43.7)

[a] *without C44 and C77–C79; CI, confidence interval; All rates are predictive margins (predicted cases per 100,000 residents) from multilevel Poisson regression models, weighted according to the 2013 European Standard Population.*

SUPPLEMENTARY TABLE S3 – Absolute and relative inequalities in cancer incidence among men and women by district-level socioeconomic deprivation

	Simple measures			Summary indices		
	**SRD (95% CI)**	**SRR (95% CI)**	***p*-value**	**SII (95% CI)**	**RII (95% CI)**	***p*-value**
**All cancers (C00–C97)[a]**						
Total	12.9 (0.0–25.9)	1.02 (1.00–1.05)	0.051	21.0 (2.1–39.8)	1.04 (1.00–1.07)	0.029
Men	47.1 (29.7–64.6)	1.07 (1.05–1.10)	<0.001	71.3 (47.8–94.9)	1.11 (1.07–1.15)	<0.001
Women	-4.5 (-18.3–9.4)	0.99 (0.96–1.02)	0.526	-7.5 (-26.4–11.5)	0.98 (0.95–1.02)	0.440
**Upper respiratory tract (C00–C06, C09–C14, C32)**						
Total	3.3 (2.1–4.5)	1.19 (1.12–1.27)	<0.001	5.0 (3.6–6.4)	1.30 (1.21–1.40)	<0.001
Men	6.6 (4.5–8.6)	1.24 (1.16–1.33)	<0.001	9.8 (7.3–12.2)	1.38 (1.28–1.50)	<0.001
Women	0.5 (-0.3–1.3)	1.06 (0.96–1.17)	0.246	0.4 (-0.5–1.3)	1.05 (0.94–1.17)	0.403
**Esophagus (C15)**						
Total	1.0 (0.4–1.5)	1.13 (1.05–1.21)	0.001	1.1 (0.4–1.7)	1.14 (1.05–1.24)	0.001
Men	2.3 (1.3–3.2)	1.19 (1.11–1.28)	<0.001	2.5 (1.4–3.6)	1.21 (1.11–1.31)	<0.001
Women	-0.3 (-0.7–0.1)	0.90 (0.79–1.02)	0.106	-0.3 (-0.8–0.1)	0.89 (0.77–1.04)	0.141
**Stomach (C16)**						
Total	3.6 (2.6–4.5)	1.20 (1.14–1.25)	<0.001	4.4 (3.3–5.4)	1.25 (1.18–1.31)	<0.001
Men	5.2 (3.8–6.6)	1.22 (1.16–1.29)	<0.001	6.7 (5.1–8.2)	1.29 (1.22–1.37)	<0.001
Women	2.2 (1.3–3.0)	1.17 (1.10–1.24)	<0.001	2.4 (1.5–3.3)	1.19 (1.12–1.27)	<0.001
**Colorectum (C18–C20)**						
Total	3.8 (1.1–6.5)	1.05 (1.01–1.09)	0.006	5.1 (1.8–8.5)	1.07 (1.02–1.12)	0.003
Men	6.4 (2.8–10.1)	1.07 (1.03–1.12)	0.001	8.0 (3.6–12.4)	1.09 (1.04–1.14)	<0.001
Women	1.7 (-0.9–4.3)	1.03 (0.99–1.08)	0.189	2.5 (-0.6–5.7)	1.04 (0.99–1.10)	0.115
**Liver (C22)**						
Total	0.5 (-0.3–1.3)	1.05 (0.97–1.13)	0.249	0.6 (-0.3–1.6)	1.06 (0.97–1.16)	0.190
Men	0.3 (-1.2–1.7)	1.02 (0.93–1.11)	0.714	0.4 (-1.3–2.1)	1.03 (0.92–1.14)	0.632
Women	0.8 (0.3–1.3)	1.16 (1.06–1.27)	0.001	0.8 (0.3–1.4)	1.17 (1.06–1.29)	0.003
**Pancreas (C25)**						
Total	0.6 (-0.2–1.4)	1.03 (0.99–1.07)	0.121	0.9 (-0.1–1.8)	1.04 (1.00–1.09)	0.067
Men	1.5 (0.3–2.7)	1.07 (1.01–1.12)	0.015	1.9 (0.6–3.3)	1.09 (1.03–1.16)	0.005
Women	-0.3 (-1.1–0.5)	0.98 (0.94–1.03)	0.477	-0.4 (-1.3–0.5)	0.98 (0.93–1.03)	0.379
**Lung (C33–C34)**						
Total	11.6 (8.0–15.2)	1.21 (1.14–1.28)	<0.001	17.8 (12.7–22.8)	1.32 (1.22–1.43)	<0.001
Men	25.6 (20.5–30.7)	1.33 (1.26–1.41)	<0.001	37.1 (30.5–43.7)	1.50 (1.40–1.61)	<0.001
Women	2.2 (-1.0–5.5)	1.06 (0.97–1.16)	0.183	2.7 (-1.7–7.0)	1.07 (0.96–1.21)	0.232
**Malignant melanoma of skin (C43)**						
Total	-6.3 (-8.3–-4.3)	0.78 (0.72–0.84)	<0.001	-10.0 (-12.6–-7.3)	0.67 (0.61–0.75)	<0.001
Men	-8.7 (-10.9–-6.5)	0.72 (0.67–0.79)	<0.001	-12.3 (-15.0–-9.5)	0.64 (0.58–0.71)	<0.001
Women	-5.8 (-8.1–-3.5)	0.78 (0.71–0.86)	<0.001	-8.3 (-11.3–-5.4)	0.71 (0.63–0.80)	<0.001
**Breast (C50)**						
Total	-	-		-	-	
Men	-	-		-	-	
Women	-7.4 (-13.4–-1.4)	0.95 (0.92–0.99)	0.016	-12.0 (-19.6–-4.4)	0.93 (0.88–0.97)	0.002

SUPPLEMENTARY TABLE S3 (continued)

	Simple measures			Summary indices		
	**SRD (95% CI)**	**SRR (95% CI)**	**p-value**	**SII (95% CI)**	**RII (95% CI)**	**p-value**
**Cervix uteri (C53)**						
Total	-	-		-	-	
Men	-	-		-	-	
Women	1.3 (0.4–2.2)	1.12 (1.03–1.21)	0.005	2.0 (1.0–3.0)	1.19 (1.09–1.30)	<0.001
**Uterus (C54–C55)**						
Total	-	-		-	-	
Men	-	-		-	-	
Women	0.1 (-1.3–1.5)	1.00 (0.95–1.06)	0.881	0.3 (-1.3–1.8)	1.01 (0.95–1.08)	0.737
**Ovary (C56)**						
Total	-	-		-	-	
Men	-	-		-	-	
Women	-1.2 (-2.2–-0.2)	0.93 (0.87–0.99)	0.016	-1.7 (-2.9–-0.6)	0.90 (0.84–0.97)	0.003
**Prostate (C61)**						
Total	-	-		-	-	
Men	4.3 (-3.6–12.2)	1.03 (0.98–1.07)	0.286	2.6 (-7.7–12.9)	1.02 (0.96–1.08)	0.623
Women	-	-		-	-	
**Testis (C62)**						
Total	-	-		-	-	
Men	0.4 (-0.4–1.3)	1.04 (0.96–1.13)	0.325	0.7 (-0.3–1.7)	1.07 (0.97–1.18)	0.167
Women	-	-		-	-	
**Kidney (C64)**						
Total	3.7 (2.6–4.8)	1.21 (1.14–1.29)	<0.001	5.1 (3.7–6.4)	1.30 (1.22–1.40)	<0.001
Men	5.0 (3.3–6.6)	1.21 (1.14–1.29)	<0.001	6.3 (4.3–8.3)	1.28 (1.18–1.38)	<0.001
Women	3.2 (2.2–4.1)	1.28 (1.19–1.38)	<0.001	4.1 (3.1–5.2)	1.39 (1.28–1.51)	<0.001
**Bladder and other urinary sites (C65–C68)**						
Total	3.6 (2.1–5.1)	1.18 (1.10–1.26)	<0.001	4.9 (3.1–6.7)	1.24 (1.15–1.34)	<0.001
Men	5.9 (3.5–8.3)	1.19 (1.10–1.27)	<0.001	7.9 (5.0–10.8)	1.25 (1.15–1.36)	<0.001
Women	1.7 (0.8–2.6)	1.18 (1.08–1.28)	<0.001	1.8 (0.8–2.8)	1.19 (1.08–1.31)	<0.001
**Bladder (C67)**						
Total	3.3 (2.0–4.6)	1.19 (1.11–1.27)	<0.001	4.5 (2.9–6.1)	1.26 (1.16–1.37)	<0.001
Men	5.5 (3.3–7.7)	1.20 (1.11–1.29)	<0.001	7.4 (4.7–10.0)	1.27 (1.17–1.39)	<0.001
Women	1.4 (0.6–2.2)	1.18 (1.08–1.29)	<0.001	1.6 (0.7–2.4)	1.21 (1.09–1.34)	<0.001
**Other urinary sites (C65, C66, C68)**						
Total	0.3 (-0.1–0.6)	1.09 (0.98–1.22)	0.111	0.3 (-0.1–0.7)	1.09 (0.96–1.23)	0.183
Men	0.2 (-0.3–0.8)	1.06 (0.93–1.21)	0.406	0.3 (-0.4–0.9)	1.06 (0.92–1.23)	0.414
Women	0.3 (0.0–0.5)	1.14 (0.99–1.31)	0.074	0.2 (-0.1–0.5)	1.10 (0.94–1.28)	0.229
**Bladder (C67, D09.0, D41.4)**						
Total	4.7 (2.5–6.9)	1.14 (1.07–1.21)	<0.001	6.5 (3.7–9.3)	1.19 (1.11–1.29)	<0.001
Men	8.1 (4.5–11.7)	1.15 (1.08–1.23)	<0.001	11.2 (6.6–15.8)	1.21 (1.12–1.31)	<0.001
Women	1.9 (0.7–3.1)	1.14 (1.05–1.23)	0.001	2.1 (0.7–3.4)	1.15 (1.05–1.26)	0.003

SUPPLEMENTARY TABLE S3 (continued)

	Simple measures			Summary indices		
	**SRD (95% CI)**	**SRR (95% CI)**	**p-value**	**SII (95% CI)**	**RII (95% CI)**	**p-value**
**Bladder and other urinary sites (C65–C68, D09.0, D09.1, D41.4, D41.7)**						
Total	5.1 (2.7–7.4)	1.14 (1.07–1.21)	<0.001	6.8 (3.7–9.9)	1.18 (1.10–1.27)	<0.001
Men	8.8 (4.9–12.7)	1.15 (1.08–1.22)	<0.001	11.8 (6.8–16.7)	1.20 (1.11–1.30)	<0.001
Women	2.1 (0.8–3.4)	1.13 (1.05–1.22)	0.001	2.3 (0.8–3.8)	1.14 (1.05–1.25)	0.003
**Central Nervous System (C70–C72)**						
Total	0.1 (-0.4–0.6)	1.01 (0.96–1.07)	0.648	0.1 (-0.5–0.6)	1.01 (0.94–1.07)	0.846
Men	-0.1 (-0.8–0.6)	0.99 (0.93–1.06)	0.817	-0.1 (-0.8–0.7)	0.99 (0.92–1.07)	0.848
Women	0.2 (-0.3–0.8)	1.03 (0.96–1.11)	0.437	0.1 (-0.5–0.7)	1.01 (0.93–1.10)	0.781
**Thyroid gland (C73)**						
Total	-2.4 (-3.2–-1.6)	0.73 (0.66–0.81)	<0.001	-3.9 (-4.8–-2.9)	0.60 (0.53–0.68)	<0.001
Men	-2.2 (-2.8–-1.6)	0.64 (0.57–0.72)	<0.001	-2.8 (-3.4–-2.1)	0.56 (0.49–0.64)	<0.001
Women	-3.0 (-4.2–-1.7)	0.76 (0.68–0.85)	<0.001	-5.1 (-6.5–-3.6)	0.62 (0.54–0.70)	<0.001
**Lymphoid/hematopoietic neoplasms (C81–C96)**						
Total	4.2 (2.0–6.3)	1.10 (1.05–1.15)	<0.001	4.7 (2.1–7.3)	1.11 (1.05–1.18)	<0.001
Men	5.0 (2.2–7.9)	1.10 (1.04–1.16)	0.001	5.5 (2.1–9.0)	1.11 (1.04–1.18)	0.002
Women	3.8 (2.0–5.7)	1.11 (1.06–1.17)	<0.001	4.2 (2.0–6.5)	1.12 (1.06–1.20)	<0.001
**Other cancers**						
Total	3.5 (1.3–5.6)	1.08 (1.03–1.14)	0.001	5.1 (2.5–7.7)	1.12 (1.06–1.19)	<0.001
Men	3.9 (1.3–6.6)	1.08 (1.03–1.15)	0.004	5.6 (2.4–8.7)	1.12 (1.05–1.20)	<0.001
Women	3.5 (1.2–5.8)	1.09 (1.03–1.15)	0.003	4.5 (1.7–7.2)	1.12 (1.04–1.19)	0.001

[a] without C44 and C77–C79; SRD, standardized rate difference (most vs. least deprived quintile); SRR, standardized rate ratio (most vs. least deprived quintile); SII, Slope index of inequality; RII, relative index of inequality; CI, confidence interval.
All measures are age-standardized according to the 2013 European Standard Population.
